# Joint influence of small-effect genetic variants on human longevity

**DOI:** 10.18632/aging.100191

**Published:** 2010-08-26

**Authors:** Anatoliy I. Yashin, Deqing Wu, Konstantin G. Arbeev, Svetlana V. Ukraintseva

**Affiliations:** ^1^ Center for Population Health and Aging, Duke University, Durham, NC 27708-0408, USA; ^2^ Duke Comprehensive Cancer Center, Duke University, Durham, NC 27708-0408, USA

**Keywords:** longevity, genome-wide association studies, aging, Framingham Heart Study

## Abstract

The results of genome-wide association studies of complex traits, such as life span or age at onset of chronic disease, suggest that such traits are typically affected by a large number of small-effect alleles. Individually such alleles have little predictive values, therefore they were usually excluded from further analyses. The results of our study strongly suggest that the alleles with small individual effects on longevity may jointly influence life span so that the resulting influence can be both substantial and significant. We show that this joint influence can be described by a relatively simple “genetic dose - phenotypic response” relationship.

The genome wide association studies (GWAS) were introduced to perform exhaustive analyses of genetic influence on complex traits. A number of recent publications emphasize that the approach did not entirely meet the expectations: Although GWAS provided important insights in genetics of particular disorders [1], it failed to detect a major portion of genetic influence on traits of interest [1-5]. In most cases genetic variants found in GWAS cannot explain heritability estimates calculated for such traits in the pre-genomic era. An important conclusion emerged from many such studies was that the complex traits are typically affected by a large number of common alleles, each of little predictive value, with small or statistically non-significant effect [1-5]. Recent suggestion to focus on the search for rare alleles with significant phenotypic effects in small population subgroups [6] requires new SNP data with minor allele frequencies (MAF) less than 1%. (Traditional GWAS deal will MAF >1%). More results could be obtained by sequencing selected areas of the genome [7,8]

In this paper we show that the use of extended approach to GWAS allows for addressing the issues of lost genetic influence on complex traits by analysing regularities of joint action of many small-effect-low-significance alleles. Using longevity trait as an example we show that the results of our analyses bring important insights into mechanisms of genetic regulation of this trait. In this approach we hypothesized that value of the complex trait (life span) depends on *number* of the small-effect “longevity” alleles, contained in individual genomes and tested this hypothesis using genome wide data on 550K SNPs from the original cohort of the Framingham Heart Study (FHS). The results show that the joint influence of small-effect alleles on life span is both significant and substantial and can be described as the “genetic dose - phenotypic response” relationship. The existence of such relationship brings a new perspective to GWAS of complex traits and can at least partly justify sizable efforts and resources that have recently been invested in GWAS.

We evaluated associations between 550,000 SNPs and life spans in 1,173 genotyped participants of the Framingham Heart Study (FHS) original cohort. After performing a standard quality control procedure [9], (call rate ≥80%; MAF>1%; HWE > 10^-7^) for each SNPs we evaluated parameters of the linear regression model by considering individuals' life span as function of SNP genotype (categorical variables) using code “0” for homozygote with respect to the major allele; “1” for heterozygote; and 2 for homozygote with respect to the minor allele. The SAS program SAS PROC REG (© SAS Institute, Inc.) has been used for this purpose. The SNPs for which the estimate of the slope parameter was positive and had p≤10^-6^ were selected as “longevity” SNPs. Note that this threshold is larger than 10^-7^ used in traditional GWAS with correction for multiple comparisons in data samples of similar size. This procedure resulted in selection of 169 “longevity” SNPs.

To evaluate joint effect of genetic variants on life span, we calculated the number of longevity SNPs (from selected set of 169 SNPs) contained in the genome of each individual in the study and performed regression analyses considering lifespan as a linear function of the number of longevity SNPs contained in person's genome. The estimates of both the intercept and the slope were positive and highly statistically significant (Figure 1).

**Figure 1.**
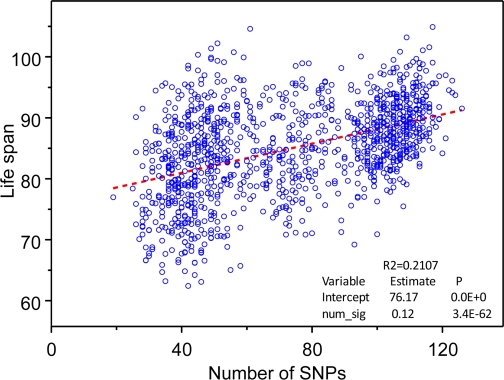
The “genetic dose - phenotypic response” relationship between the numbers of selected 169 longevity alleles contained in individuals' genome and mean life span obtained in the analyses of 550K SNP data on participants of the original FHS cohort. Regression analyses were performed using SAS PROC REG (© SAS Institute, Inc.) with correction for heteroscedasticity.

The estimated dependence explained 21% of variance in life span. This estimate seems to be reasonable if one takes into account that narrow sense heritability in life span is estimated at the level about 25% [10]. The estimated relationship between life span and the number of longevity SNPs shown in Figure 1 is the main result of this paper. It shows that in studies of genetic determinants of longevity the joint influence of many small-effect genetic variants may be substantial. We suggest that similar “genetic dose” - “phenotypic response” relationship is likely to characterize genetic influence on many other complex traits.

The two aspects of performed analyses require additional testing. The first is the use of data on all genotyped individuals from the original FHS cohort, which include first degree relatives from 618 families. The second is the fact that the two procedures: (i) selection of longevity SNPs and (ii) testing the presence of their joint influence on life span used data on the same individuals. To check whether the exclusion of relatives from the list of study subjects modifies the results of analyses, we randomly selected 618 individuals, one from each family, identified a set of “longevity” SNPs using the procedure described above, and estimated dependence of life span on the number of selected longevity SNPs in these individuals. To diminish the effect of sampling, we repeated this procedure 10 times. In each such analysis, the estimates of slope and intercept were positive and highly statistically significant with p≤10^-19^. These results suggest that the conclusion about joint influence of longevity SNPs on life span does not depend on the presence or absence of relatives among the study subjects. To take into account variants selected in each experiment, we unified sets of longevity SNPs selected in each of 10 experiments. This procedure resulted in the set with 70 genetic variants. Note that the reduction in the number of study subjects (because of excluding genetically dependent individuals) increases the chances of selecting false positive variants. To diminish the number of such variants, we intersected the set of 70 SNPs with the set of 169 SNPs, selected earlier using data on the entire FHS cohort. This procedure resulted in 39 longevity SNPs.

This set of 39 SNPs was then used in regression analyses where life span was considered as a linear function of the number of longevity SNPs contained in person's genome. The result is shown in Figure 2.

**Figure 2.**
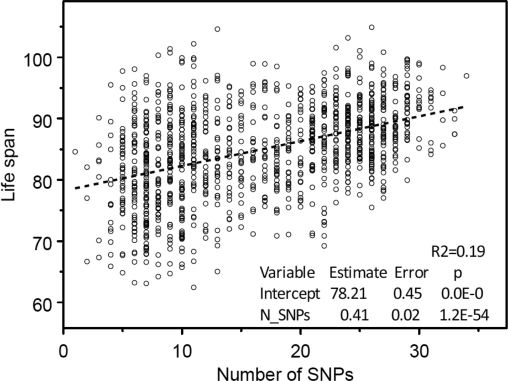
The “genetic dose - phenotypic response” relationship between the numbers of selected 39 longevity alleles contained in individuals' genome and mean life span obtained in the analyses of 550K SNP data on participants of the original FHS cohort. Regression analyses were performed using SAS PROC REG (© SAS Institute, Inc.) with correction for heteroscedasticity.

One can see from this figure that the estimates of both the intercept and slope are statistically significant. The Figure 3 shows no dependence of life span from the number of SNPs taken randomly from the pool of SNPs without 39 selected longevity SNPs.

**Figure 3.**
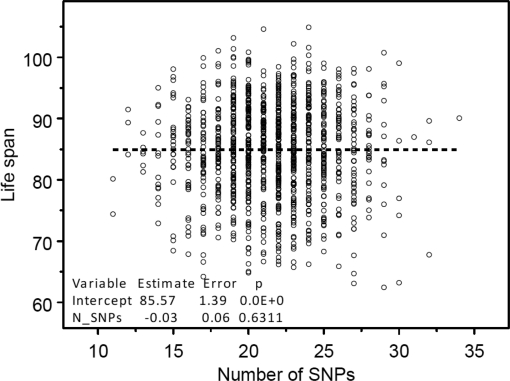
The absence of dependence between the numbers of randomly selected 39 genetic variants contained in individuals' genome and life span. These genetic variants were randomly selected from the same pool of SNPs excluding longevity alleles. Regression analyses were performed using SAS PROC REG (© SAS Institute, Inc.) with correction for heteroscedasticity.

The analyses showed that the estimates of both the intercept and slope are highly statistically significant. The estimated dependence of life span on genes explains 19% of variance in life span, which is close to 21% estimated earlier. Thus, the presence of relatives in the population used for selecting longevity SNPs does not affect the conclusion about the presence of “genetic dose” - “phenotypic response” relationship. The fact that 39 selected SNPs explained almost the same percent of life span variance as 169 SNPs selected earlier (19% vs 21%) indicates that this set of SNPs deserves further analyses. Table 1 shows how selected SNPs are related to known genes.

**Table 1.** Summary characteristics of the 39 SNPs revealed in the study and gene/protein functions for closest genes (known or suggested).

SNP rs#	Chr #	Position	Ancestral allele	Type	Distance to gene	Closest gene	Gene full name	Gene/protein function
rs2031577	10	4050003	G	INTERGENIC	-17129	RP11-433J20.2	H. sapiens chr 10 clone RP11-433J20	
rs6489785	12	121363724	C	INTERGENIC	-52622	HNF1A (TCF1)	HNF1 homeobox A	liver transcription factor
rs3847687	12	131525053	T	INTRONIC	0	GPR133	G protein-coupled receptor 133	transmembranic signal transduser; activates G proteins within cell
rs4891159	18	74101941	G	INTRONIC	0	ZNF516	zinc finger protein 516	the part of transcription factors
rs10445407	17	79261809	A	INTRONIC	0	SLC38A10	solute carrier family 38, member 10	amino acid transporter
rs4745062	9	73784264	C	INTRONIC	0	TRPM3	transient receptor potential channel	mediates calcium entry potentiated by calcium store depletion
rs2024714	20	60212494	C	INTRONIC	0	CDH4	R-cadherin (retinal)	calcium-dependent cell-cell adhesion
rs7315621	12	132085196	G	INTERGENIC	-60412	AC117500.2		
rs16975963	19	38325536	G	NON CODING GENE	0	AC016582.2		
rs4732038	7	134250322	C	INTRONIC	0	AKR1B15	aldo-keto reductase family 1, member B15	superfamily of reductases that reduce aldehydes and ketones to alcohols
rs2516739	16	2097158	N/A	INTRONIC	0	NTHL1	nth endonuclease III-like 1	base excision repair; DNA N-glycosylase of the endonuclease III family
rs7874142	9	137704782	A	INTRONIC	0	COL5A1	collagen, type V, alpha 1	regulates the assembly of heterotypic fibers in tissues
rs4468878	20	59928237	C	INTRONIC	0	AL365229.1	near CDH4	possibly cell-cell adhesion
rs13008689	2	8530256	G	INTERGENIC	-153466	AC011747.3		
rs2273	4	76889388	C	INTRONIC	0	SDAD1	SDA1 domain containing protein	preferentially expressed in fetal tissues
rs2882281	13	90622455	C	INTERGENIC	-21630	RP11-388D4.1	locus tag for a pseudogene	
rs2282032	14	90758891	G	INTRONIC	0	C14orf102	chromosome 14 open reading frame 102	
rs9876781	3	48487338	A	NON CODING GENE	0	RP11-24C3.2		
rs6568433	6	106829537	C	INTERGENIC	-39044	AL109920.3		
rs9517320	13	99126303	A	INTRONIC	0	STK24	serine/threonine kinase 24	participates in the mitogen-activated protein kinase (MAPK) cascade
rs4148546	13	95680285	G	INTRONIC	0	ABCC4	ATP-binding cassette, sub-family C (CFTR/MRP)	ATP-binding cassette (ABC) transporter
rs9592783	13	71883214	G	INTERGENIC	-128884	DACH1	dachshund homolog 1 (Drosophila)	a chromatin-associated protein that regulates gene expression and cell fate; highly conserved
rs739401	11	3036324	T	INTRONIC	0	CARS	cysteinyl-tRNA synthetase	catalyzes the aminoacylation of a tRNA;
rs10256972	7	1039003	C	INTRONIC	0	C7orf50	chromosome 7 open reading frame 50	
rs3212335	15	27012141	C	INTRONIC	0	GABRB3	GABA A receptor, beta	ionic channel family that serves as the receptor for GABA; may be associated with memory
rs6915183	6	166706169	G	INTERGENIC	-12999	PRR18	proline rich 18	
rs4721135	7	1912222	G	INTRONIC	0	MAD1L1	MAD1 mitotic arrest deficient-like 1	component of the mitotic spindle-assembly checkpoint
rs3106598	13	61678912	G	INTERGENIC	-304909	PCDH20	protocadherin 20	transmembrane receptor, a role in specific cell-cell connections in the brain
rs1356888	2	50516018	C	INTRONIC	0	NRXN1	cell adhesion in nervous system	
rs9616906	22	51104680	G	UPSTREAM	-3552	AC000050.2		
rs13053175	22	37613309	T	UPSTREAM	-7992	RAC2	ras-related C3 botulinum toxin substrate 2	GTPase of the RAS superfamily regulating cell growth, cytoskelet, and the protein kinases activation
rs5766691	22	47532396	G	INTRONIC	0	TBC1D22A	TBC1 domain family	
rs13118159	4	1365127	N/A	INTRONIC	0	RP11-1244E8.1		
rs7168365	15	53805825	C	DOWNSTREAM	-113	WDR72	WD repeat domain 72	
rs7493138	14	29021928	C	INTERGENIC	-213122	FOXG1	forkhead box G1	transcription factors
rs432203	2	70764688	A	INTRONIC	0	TGFA	transforming growth factor, alpha	competes with EGF for binding to the EGF receptor
rs6813479	4	137660383	A	INTERGENIC	-57494	RP11-138I17.1		
rs1327533	9	113131163	T	INTRONIC	0	SVEP1	EGF and pentraxin domain containing 1	
rs2826891	21	22910116	T	INTRONIC	0	NCAM2	neural cell adhesion molecule 2	brain protein, superfamily of the immunoglobulin

*Enrichment with genes related to cell-cell adhesion can be noticed. Since cell-cell adhesion proteins play crucial role in cell sensitivity to contact inhibition and because insensitivity to contact inhibition is critical for cancer development, especially for manifestation of invasion and metastasis, we speculate that this enrichment might potentially be linked to a higher resistance to cancer among long-living individuals.

The second aspect mentioned above deals with prediction and replication. If the procedures, described above, do select longevity variants, and if the detected pattern of joint influence of such variants on life span is a property of a biological mechanism, then genetic variants selected using data on one population should be able to predict life spans in other genetically independent population of individuals who experienced similar environmental and living conditions. To test this, we randomly divided all 618 families into two groups. Data on individuals from the first 309 families plus data on 162 individuals with missing family identities were used for selecting SNPs having effect on life span. Then for each individual in the second (genetically independent) group we identified the number of such SNPs contained in person's genome. We estimated parameters of the linear regression model considering life span as function of the number of longevity variants contained in the genomes of individuals from the same (first) group and from the second (independent) group of individuals. To replicate the result, longevity SNPs selected from data on the second population were used for evaluating linear “genetic dose” - “life span response” relationship on the same population, as well as on the first population of individuals genetically independent from the second one. To reduce the sampling effect, the procedure of random division of the 618 families into two groups with subsequent selection of longevity variants and estimating regression coefficients in the “genetic dose - phenotypic response” relationship was repeated 10 times. The results are shown in Table 2.

**Table 2.** 

#	N_1_	N_2_	N_1SNP_	N_2SNP_	α_1_	α_1_*	α_2_	α_2_*
1	661	512	52	8	0.30	0.26	0.14	0.16
2	689	484	40	9	0.42	0.35	0.21	0.23
3	627	546	18	43	0.87	0.80	0.28	0.22
4	677	496	20	25	0.67	0.63	0.56	0.49
5	680	493	34	16	0.47	0.41	0.33	0.31
6	630	543	32	22	0.48	0.39	0.46	0.48
7	631	542	43	15	0.40	0.31	0.22	0.27
8	658	515	14	39	0.86	0.99	0.33	0.25
9	647	526	31	18	0.48	0.38	0.38	0.42
10	672	501	37	10	0.44	0.37	0.24	0.27

The results of 10 experiments in which genetic variants individually affecting life span (longevity SNPs) were selected twice using data on two populations representing genetically independent genotyped individuals in the original Framingham Heart Study (FHS) cohort for whom life span data are available. The longevity SNPs selected from data on the first population were used for evaluating linear “genetic dose” - “life span response” relationship on the same population, as well as on the second population of individuals. In turn, longevity SNPs selected from data on the second population were used for evaluating linear “genetic dose” - “life span response? relationship on the same population, as well as on the first population of individuals. Column “#” shows experiment's number. Columns N_1_ and N_2_ show the number of individuals in the first and in the second (genetically independent) populations. Columns N_1SNP_ and N_2SNP_ show the number of longevity SNPs selected using data on the first (original) and on the second (rest) populations respectively. Column α_1_ shows the estimate of the slope of the regression line describing dependence of life span on the number of longevity SNPs contained in the genomes of individuals from the first population. Column α_1_* shows the estimate of the slope of the regression line describing dependence between life span and the number of selected longevity SNPs contained in genomes of individuals from the second (independent) population. The estimates α_1_ and α_1_* use SNPs selected in the analyses of connection between SNPs and life span in the first (original) population. Column α_2_ shows the estimate of the slope of the regression line describing dependence of life span on the number of longevity SNPs contained in the genomes of individuals from the second population. Column α_2_* shows the estimate of the slope of the regression line describing dependence between life span and the number of selected longevity SNPs contained in genomes of individuals from the first population. The estimates α_2_ and α_2_* use SNPs selected in the analyses of connection between genes and life span in the second (rest) population. All four estimates are highly significant (p<1×10^-10^).

One can see from this table that the effect of the number of selected “longevity” SNPs on life span is significant in both groups. These analyses show that developed approach has predictive power, and that joint influence of longevity SNPs on life span can be replicated in populations of genetically independent individuals.

Some recent studies provide arguments that the Bonferroni corrections for multiple comparisons, traditionally used in GWAS, are too rigid and should be relaxed [11]. The results of this study support this view: many genetic variants involved in the “genetic dose - phenotypic response” relationship would not be selected by traditional GWAS methods. We found that relaxing the procedure for selecting longevity alleles (the use of selection threshold p≤10^-6^ instead of p≤10^-7^) increases the number of selected longevity SNPs having small effects and improve the fit of the life span data by the “number of longevity SNPs - life span” curve. This suggests that taking effect size of alleles into account may help reveal additional features of genetic influence on complex traits.

The possibility of using a straight line for approximating “the number of longevity SNPs -- life span” relationship indicates the presence of substantial additive component of the genetic contribution to longevity. It is relevant to note that additive genetic effects were the subject of numerous studies in quantitative genetics of the pre-genomic era. Many genetic calculations (e.g., estimates of narrow sense heritability of complex traits) were based on the assumption about the additive nature of genetic component of phenotypic variation. The availability of genome-wide data nowadays allows for evaluating such effects directly. Moreover, evaluating the non-additive (non-linear) joint genetic influence (epistasis) becomes also possible with the use of more sophisticated patterns of the “dose - response” relationship.

While the replication of findings became a standard requirement in GWAS, the results of our analyses suggest that in studying joint effect of many alleles this practice needs to be revised. Our analyses show that one should not expect that exactly the same sets of genetic variants will contribute to “genetic dose - phenotypic response” relationship evaluated using data on other population. One reason for this may be gene-environment interaction: difference in populations' exposure to external conditions is likely to produce difference in genetic regulation of the trait in these populations. Identification of genetic variants “sensitive” to specific external signals will open new opportunities for studying the role of genetic and non-genetic factors in complex traits.
